# The Impact of Armed Conflicts on the Prevalence, Transmission, and Management of Infectious Diseases: A Systematic Review

**DOI:** 10.7759/cureus.79450

**Published:** 2025-02-22

**Authors:** Reem Alfaleh, Wessam A Alsuwailem, Renad T Almazyad, Ftoon F Alanazi, Lujain T Alanazi

**Affiliations:** 1 Department of Laboratory, Salamaty Polyclinic, Arar, SAU; 2 National Center for the Prevention and Control of Plant Pests and Animal Diseases (WEQAA Center), Ministry of Environment, Water and Agriculture, Arar, SAU; 3 Department of Laboratory, Leen Polyclinic, Arar, SAU

**Keywords:** how infectious diseases affect population, impact of conflict on infectious disease, infectious disease, management of infectious diseases, transmission of infectious disease

## Abstract

Armed conflicts persist despite global peace efforts, driven by cultural, religious, and ethnic divisions. These conflicts significantly impact public health by exacerbating the spread of infectious diseases and disrupting essential healthcare systems. This study aimed to assess the complex relationship between conflict and the prevalence, transmission, and management of infectious diseases in the affected populations. This study followed the Preferred Reporting Items for Systematic Reviews and Meta-Analyses (PRISMA) guidelines to conduct a comprehensive search across five databases (EBSCO, PubMed, Web of Science, Cochrane, and Google Scholar) focusing on publications from 2020 to 2024. The search centered on topics such as infectious diseases, epidemics, war, health infrastructure, and public health systems. Papers published in English were screened using the Rayyan™ tool (Rayyan Systems Inc., Cambridge, MA) to ensure relevance to infectious diseases and conflicts. Data analysis was carried out using RevMan software (The Cochrane Collaboration, London, UK), while the Newcastle-Ottawa Scale (NOS) was employed to evaluate the quality of the included studies. The results reveal that conflicts significantly disrupt healthcare systems, leading to an increased prevalence of diseases such as tuberculosis, cholera, and soil-transmitted helminthiasis (STH). Effective interventions, including improved water, sanitation, and hygiene (WASH) conditions, targeted vaccination campaigns, and strengthened healthcare infrastructure, were critical in mitigating outbreaks. Despite methodological variations, the studies highlighted the multifaceted impact of conflict on public health. Conflict creates complex interdependencies between environmental, social, and health factors, worsening disease prevalence and management. In order to improve WASH conditions, prevent diseases, guarantee medication supply, and improve healthcare, coordination of efforts is essential. Future studies should examine community resiliency, socioeconomic determinants, and intervention evaluation.

## Introduction and background

Despite the absence of major wars in the world, armed conflicts remain a harsh reality in many regions, fueled by deep-rooted ethnic, cultural, and religious divisions [1]. These conflicts leave a profound mark on public health, intensifying the spread and impact of infectious diseases. Armed conflicts have been associated with the destruction of critical health infrastructure and systems [1,2]. Communities exposed to conflicts often grapple with periodic outbreaks of armed violence that lead to loss of lives and property and a sustained conflict that may last for months to years, further jeopardizing their access to critical healthcare [2]. The long-term consequences of sustained conflicts are poor access to health services, which exacerbate the spread of otherwise preventable diseases. The destruction of critical health infrastructure and forced migration from one area to another in search of safety are the primary contributors to the spread of infectious diseases among populations exposed to conflicts [2,3].

According to a recent study by Dattani et al., although conflict ranks 27th among the causes of death globally, violent conflicts are the ninth leading cause of death among people aged 15-49 years [4]. While infectious diseases only account for about 30% of global mortality these days, their prevalence is exacerbated by armed conflicts that disrupt public health interventions that are already in a poor state in these regions [1,4]. Research shows that in addition to the destruction of healthcare infrastructure, many healthcare providers (HCPs) lose their lives in these conflicts, especially during their response efforts to attend to afflicted victims [5]. The overall impact of conflicts and their association with infectious disease prevalence weakens the public health systems, rendering them incapable of implementing effective disease control programs and responding to public health emergencies [2,4-6].

Disease outbreaks in areas affected by conflicts pose distinct difficulties to public health and emergency response efforts. Timely detection and response require a functional health system to provide sufficient resources, human or otherwise, to combat the spread of infections [7]. The destruction of such response systems derails the detection, response, and containment of infections, further prolonging the suffering of the affected population, in addition to increasing the spread of such infections to other regions as they run for safety.

A stark reminder of this is the ongoing Russia-Ukraine war that broke out in 2022 during the post-pandemic phase of the COVID-19 pandemic. The war has resurfaced a discourse on the dangers of infectious disease outbreaks among displaced communities and underscored the critical need for effective public health emergency preparedness and response strategies to address infectious disease spread in and around war zones [8]. According to Essar et al., as of February 2022, the war in Ukraine had exacerbated the risk of infectious disease outbreaks in the country. The destruction of critical health infrastructure led to a rise in the prevalence of chronic infections such as tuberculosis and human immunodeficiency virus (HIV)/acquired immunodeficiency syndrome (AIDS) in Ukraine [7]. Furthermore, overcrowding in refugee camps, poor sanitation, malnutrition, and lack of access to clean water intensify the vulnerability of affected populations to infectious disease outbreaks.

However, evidence of the actual extent of conflict on infectious disease is largely unknown. As evidenced by Goniewicz et al., the impact of infectious disease on the military is well-documented throughout history, with more soldiers dying from infectious diseases than from combat during deployment to war-torn regions [3]. This systematic review, therefore, aims to explore the complex relationship between conflict and the prevalence, transmission, and management of infectious diseases in the affected populations.

## Review

Materials and methods

Literature Search Strategy

This study followed the Preferred Reporting Items for Systematic Reviews and Meta-Analyses (PRISMA) 2020 guidelines [8] to conduct a comprehensive search across five medical databases for relevant scholarly publications published between 2020 and 2024. These databases include Web of Science, PubMed, Cochrane Library, EBSCO, and Google Scholar. We utilized a series of keywords tailored to each database including "infectious disease", "communicable diseases", "disease incidence", "conflict", "war", "epidemics", "emergency health response", "health infrastructure," and "public health systems."

Eligibility, Data Extraction, and Management

We used the Rayyan™ tool (Rayyan Systems Inc., Cambridge, MA) to rigorously assess the retrieved articles for eligibility by comparing them with the predefined inclusion and exclusion criteria that were agreed upon by all the researchers involved in this study.

Inclusion criteria: Studies were included if they focused explicitly on disease prevalence, transmission patterns, and healthcare disruptions in conflict-affected populations. Only original research articles, including cohort studies, case-control studies, cross-sectional analyses, and descriptive epidemiological studies, were considered to ensure a diverse range of methodological approaches. The time frame was limited to 2020-2024 to capture recent trends, particularly in the post-COVID-19 period, while only English-language articles with full-text access were included to maintain data consistency and accessibility. The selected time frame (2020-2024) was chosen to capture the most recent studies on the intersection of armed conflicts and infectious diseases, particularly in the post-COVID-19 era.

Exclusion criteria: Studies were excluded if they lacked direct relevance to infectious diseases in conflict settings, such as research conducted in stable regions or general public health studies. Review articles, meta-analyses, editorials, and commentaries were excluded to ensure that only primary research data were analyzed. Articles with poor methodological rigor, such as those lacking control groups, statistical analysis, or clear study design, were also omitted. Unpublished studies, grey literature (e.g., conference abstracts, preprints, and dissertations), and non-peer-reviewed sources were not considered to maintain the reliability of findings. Additionally, studies in languages other than English were excluded due to feasibility constraints in translation.

Statistical Data Analysis

Data analysis was performed using RevMan (The Cochrane Collaboration, London, UK) version 5.4.1 software. The systematic review incorporated a statistical approach to assess study quality and examine potential biases within the included studies.

Quality Assessment

The quality of the included studies was evaluated using the Newcastle-Ottawa Scale (NOS) [9]. This tool assessed the risk of bias across three domains: selection, comparability, and outcome. Studies were classified as having a high, moderate, or low risk of bias based on predefined scoring thresholds as follows: high bias, studies scoring 0-3; moderate bias, studies scoring 4-6; and low bias, studies scoring 7-9. This scoring framework was explicitly defined to enhance transparency and reproducibility.

Results

**Figure 1:**
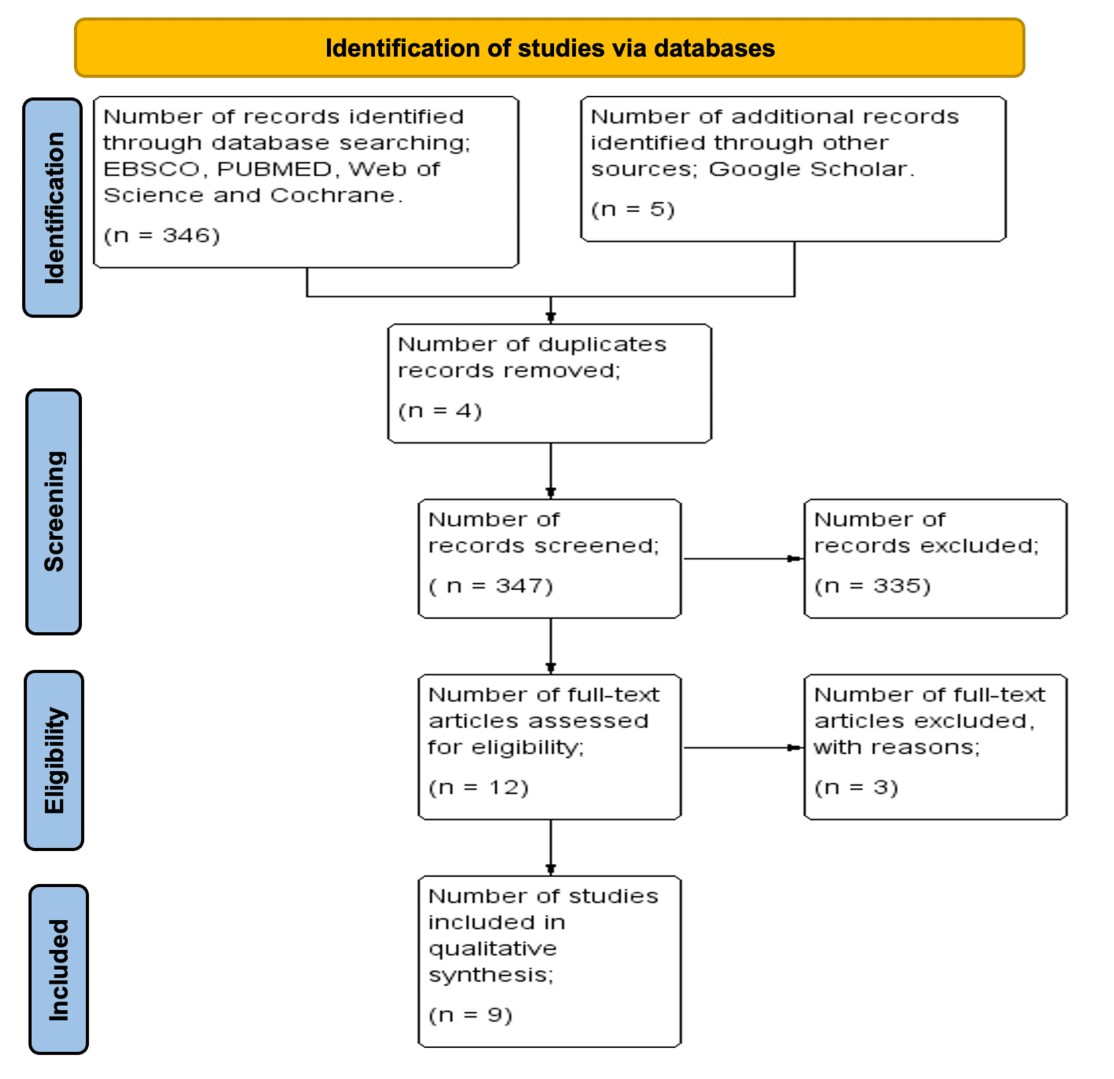
PRISMA flow diagram PRISMA: Preferred Reporting Items for Systematic Reviews and Meta-Analyses

Initially, 351 studies were retrieved from five electronic databases (Web of Science: 253, EBSCO: 19, PubMed: 33, Cochrane Library: 41, Google Scholar: 5). After removing 16 duplicate records, 335 studies remained for title and abstract screening. At this stage, 289 studies were excluded for reasons such as lack of relevance to conflict and infectious diseases, non-primary research (e.g., reviews and commentaries), or insufficient data. This left 46 full-text articles for detailed assessment. Following full-text screening, three studies were excluded due to incomplete or missing data (one study), duplication across multiple databases (one study), and methodological concerns such as small sample size and unclear study design (one study). As a result, nine studies met all inclusion criteria and were included in the final qualitative synthesis to assess the impact of armed conflict on the prevalence, transmission, and management of infectious diseases as illustrated in Figure 1.

Study Characteristics

The key attributes of the included studies for review are detailed in Table 1. All the studies included for review were conducted in various regions of the world and were all published in the English language.

**Table 1:** Characteristics of the included studies COVID-19: coronavirus disease 2019, TB: tuberculosis, WHO: World Health Organization, STH: soil-transmitted helminthiasis, PCR: polymerase chain reaction, WASH: water, sanitation, and hygiene, N/A: not applicable

Authors	Study design	Intervention	Sample size	Outcome/results	Conclusion
Mousavi and Anjomshoa (2020) [10]	Descriptive analytical approach study	Examination of the interplay between Yemen's ongoing war, the fragile healthcare system, and the COVID-19 outbreak, highlighting the compounded public health crises	N/A	War-related destruction has left only 50% of healthcare facilities functional. Yemen faces multiple public health challenges, including the re-emergence of cholera, diarrhea, dengue, and measles. COVID-19 risks overwhelming the under-resourced healthcare system, with projections of infecting up to 90% of the population if unaddressed.	Urgent international humanitarian action is needed to end the war, lift blockades, rebuild health infrastructure, and strengthen Yemen's health system to prevent further public health disasters.
Alashavi et al. (2021) [11]	Cross-sectional study	Implementation of TB service delivery in Northwest Syria from 2019 to 2020, including the establishment of TB centers and the introduction of diagnostic, treatment, and outreach services aligned with WHO standards	785 TB cases identified	Treatment success rates were 70.7% in Q3 2019 and 62.1% in Q4 2019. Challenges included high rates of loss to follow-up and the impact of COVID-19 on TB services. Collaborative efforts improved diagnostic capacity, including rapid molecular diagnostics and case detection.	The project successfully provided quality TB care under challenging humanitarian conditions, highlighting the need for continued expansion, better diagnostic tools, and improved adherence strategies to meet WHO TB control goals.
Geleto et al. (2022) [12]	Cross-sectional study	Assessment of STH prevalence and associated malnutrition among children under five in conflict-affected areas of southern Ethiopia	405 children under five years	STH prevalence: 67.4%; the most common parasite was Ascaris lumbricoides (90% prevalence). Malnutrition prevalence: 54.2%, with strong associations between STH infections and malnutrition (e.g., severe stunting and wasting). Key risk factors: internal displacement, lack of private latrines, limited access to clean water, and improper waste disposal practices.	STH infections and malnutrition are significant health challenges in conflict zones. Improved sanitation, access to clean water, and targeted nutritional interventions are crucial for health improvement in these populations.
Chen et al. (2023) [13]	Quantitative observational study	Analysis of malaria prevalence as a factor influencing civil conflict dynamics in Sub-Saharan Africa, using 20 years of geo-referenced conflict and malaria data	Geo-referenced grid-level data covering 20 years (2000-2019) in Sub-Saharan Africa	The study finds a negative correlation between malaria prevalence and the occurrence of civil conflicts. Areas with higher malaria prevalence have reduced odds of conflict due to deterrence by health risks.	Malaria prevalence appears to deter the frequency of militarized battles in malaria-prone areas, suggesting a nuanced relationship between infectious diseases and civil conflict dynamics in the region.
Farah et al. (2023) [14]	Descriptive epidemiological study	Description of an enhanced national communicable disease surveillance system in Lebanon, incorporating routine and syndromic surveillance systems to monitor diseases among Syrian refugees and the Lebanese population	Data including all reported cases in the Lebanese population and Syrian refugee population during the study period	Successful detection and monitoring of outbreaks of viral hepatitis A, leishmaniasis, mumps, and measles among Syrian refugees and the Lebanese population. A decrease in hepatitis A incidence after 2014, attributed to improved WASH conditions. The incidence of leishmaniasis decreased from 205 per 100,000 in 2013 to less than 12 per 100,000 by 2016 due to enhanced treatment and monitoring. Mumps outbreaks in 2014-2015 were addressed with enhanced routine vaccination campaigns. Measles outbreaks were managed with targeted immunization drives.	The surveillance system rose to the challenge of monitoring health in a large, displaced population, enabling the early detection and control of disease outbreaks. Its success relied on strong collaboration with international partners, a flexible design that could adapt to changing needs, and carefully targeted interventions. The study highlights the importance of flexible surveillance systems for emergency contexts and the need for experience-sharing across countries facing similar challenges.
Petakh et al. (2024) [15]	Descriptive epidemiological study	Surveillance of human leptospirosis infections in Ukraine from 2018 to 2023, focusing on environmental and social factors influencing incidence rates	1,384 cases reported over the six-year period, with a detailed analysis of 433 cases in 2023	In 2023, leptospirosis cases reached their highest level, with a notification rate of 1.06 per 100,000, with Zakarpattia Oblast reporting the highest incidence at 12.08 per 100,000. The surge in cases was driven by a combination of factors, including flooding, excessive humidity, and a rise in rodent populations, problems worsened by the conflict and widespread damage to infrastructure. Incidence among adults was consistently higher than among children.	The study underscores the need for comprehensive public health measures in Ukraine, including improved surveillance, rodent control, vaccination, and public education, to address the growing leptospirosis burden in conflict-affected and flood-prone areas.
Chamankar (2024) [16]	Retrospective study	Analysis of the health impacts of World War II on the Persian Gulf, focusing on the effects of Allied occupation on public health, infectious disease outbreaks, and healthcare resource shortages	N/A (historical study based on archival documents, newspapers, and publications)	The study revealed how deeply public health suffers in the face of poverty, malnutrition, and outbreaks of infectious diseases such as smallpox, malaria, and typhus. These challenges are made even worse by drug shortages and the chaos caused by war and occupation, leaving communities struggling to cope with basic health needs.	The Allied occupation during World War II severely affected public health in Iran's southern and southeastern regions, leading to critical shortages in medicines, healthcare facilities, and sanitation, with widespread outbreaks of communicable diseases.
Petakh et al. (2024) [17]	Descriptive epidemiological study	Analysis of communicable disease trends in Ukraine during 2018-2023, with a focus on the impact of the Russian invasion, the COVID-19 pandemic, and environmental disruptions	National-level data from Ukraine's comprehensive disease surveillance system	Decrease in salmonellosis, shigellosis, and rotavirus incidence. Increase in viral hepatitis A, chronic hepatitis B, chronic hepatitis C, and tuberculosis incidence in 2023. Significant increase in whooping cough cases in 2023 due to disrupted vaccination efforts. The rise in respiratory and diarrheal diseases is linked to war-induced conditions such as population displacement and infrastructure damage.	The study sheds light on how war and the pandemic have deeply strained Ukraine's healthcare system, underscoring the urgent need for ongoing vaccination programs, international aid, and stronger disease monitoring to tackle the growing public health challenges.
Zinszer and Abuzerr (2024) [18]	Cross-sectional study	Analysis of WASH insecurity and its impact on infectious disease outbreaks among internally displaced populations in Gaza	Quantitative survey of 1,500 displaced households and qualitative data from 15 focus group discussions and key informant interviews	High incidence rates of infectious diseases among displaced populations, particularly acute respiratory infections (49.3%) and diarrhea (24.9%). WASH insecurity factors such as limited water access, shared latrines, and lack of hygiene supplies were significantly associated with health risks. Qualitative data highlighted severe living conditions, psychological stress, and community resilience.	Conflict-driven displacement in Gaza exacerbates WASH insecurity, leading to significant public health risks. Urgent interventions to improve water access, sanitation facilities, and hygiene supplies are critical to mitigating health risks and enhancing the well-being of displaced populations.

Assessment of Risk of Bias Items

For the assessment of the risk of bias, we utilized the Newcastle-Ottawa Scale. The total scores for the included studies ranged from 3 to 8 out of a possible 9 points, based on the assessment criteria. Studies by Alashavi et al. [11], Chen et al. [13], Farah et al. [14], and Zinszer and Abuzerr [18] had a moderate risk of bias, with scores of 5, 6, 4, and 6, respectively. A lower likelihood of bias was observed in the studies by Geleto et al. [12], Petakh et al. [15], Chamankar [16], and Petakh et al. [17], which achieved scores of 7, 8, 7, and 7, respectively. In contrast, the study by Mousavi and Anjomshoa [10] had a high risk of bias with a total score of 3 (Table 2).

**Table 2:** Newcastle-Ottawa Scale tool used in the assessment of risk of bias items of the included studies Selection: Q1. Is the exposure cohort representative? Q2. How did you select the non-exposure group? Q3. How was the exposure calculated? Q4. Evidence that there was no intended result at the beginning of the study. Comparability: Q5. Based on the design or analysis, is the cohort comparable? Results: Q6. Assessment of the outcome. Q7. Did the duration of the follow-up allow for the emergence of results? Q8: Is the follow-up for the cohort sufficient? The methodological quality was assessed using the Newcastle Ottawa Scale, which comprises eight questions divided into four categories (selection, ascertainment, causation, and reporting). A score of 7-9 indicates high-quality research with low risk, fair-quality research with moderate risk was given a score of 4-6, and low-quality study with the highest risk of bias was given a score of 0-3.

Authors	Section				Comparability	Outcome			Total quality score	Likelihood of bias
	Q1	Q2	Q3	Q4	Q5	Q6	Q7	Q8		
Mousavi and Anjomshoa (2020) [10]	*	-	-	*	-	*	-	-	3	High risk
Alashavi et al. (2021) [11]	*	-	*	-	-	*	*	*	5	Moderate risk
Geleto et al. (2022) [12]	*	-	*	*	*	*	*	*	7	Low risk
Chen et al. (2023) [13]	*	-	*	-	*	*	*	*	6	Moderate risk
Farah et al. (2023) [14]	*	-	-	*	-	*	*	-	4	Moderate risk
Petakh et al. (2024) [15]	*	*	*	*	*	*	*	*	8	Low risk
Chamankar (2024) [16]	*	*	*	*	-	*	*	*	7	Low risk
Petakh et al. (2024) [17]	*	*	*	*	-	*	*	*	7	Low risk
Zinszer and Abuzerr (2024) [18]	*	*	*	*	-	*	*	-	6	Moderate risk

Most studies scored 2 or 3 in the selection domain, indicating reasonably representative exposure cohorts, but none scored in the comparability domain, highlighting this as the most common methodological limitation. Furthermore, the outcome assessment and duration of follow-up varied, affecting the total quality scores across studies. Overall, the methodological quality of the included studies was moderate. Therefore, while these findings provide valuable insights, caution should be exercised in their interpretation due to the identified limitations.

Discussion

Despite global peace, armed conflicts continue to arise due to cultural, religious, and ethnic divisions. These conflicts have a substantial influence on public health, exacerbating infectious diseases and devastating key healthcare infrastructure. Historically, infectious diseases have killed more soldiers than combat in conflict zones. This systematic review, therefore, aimed to explore the complex relationship between conflict and the prevalence, transmission, and management of infectious diseases in affected populations.

According to a study conducted by Mousavi and Anjomshoa, only half of Yemen's healthcare facilities are operational, highlighting the country's dire situation. The recurrence of diseases such as cholera and COVID-19, coupled with war-related destruction, intensifies this dilemma [10]. Moreover, as noted by Alashavi et al., COVID-19 disruptions and high follow-up loss were major factors in the drop in TB treatment success rates, which declined from 70.7% in Q3 2019 to 62.1% in Q4 2019 [11]. Furthermore, Geleto et al. discovered a growing awareness of serious public health risks associated with soil-transmitted helminthiasis (STH), which has a staggering incidence rate of 67.4%. The most common risk included the parasite *Ascaris lumbricoides*, found in 90% of infected individuals. Additionally, malnutrition, particularly severe stunting and wasting, which has a 54.2% prevalence, was found to have a clear relationship with STH infections [12].

The study's findings provide a complex picture of public health dynamics in regions affected by war and environmental problems. The study by Chen et al. found a significant negative correlation between the prevalence of malaria and the occurrence of civil conflict. This suggests that areas with higher malaria prevalence also have lower rates of violence, possibly due to the deterrent effect of malaria-related health issues. Serious health issues may deter conflict participation, suggesting a complex link between social stability and health [13]. On the other hand, Farah et al. discovered that among Syrian refugees and Lebanese citizens, efficient public health initiatives significantly reduced occurrences of viral hepatitis A, leishmaniasis, mumps, and measles. The incidence of these diseases decreased considerably due to improved WASH conditions, focused vaccination programs, and immunization drives, highlighting the significance of prophylactic efforts [14]. Petakh et al. claim that the rise in leptospirosis reporting rates was brought on by flooding, high humidity, and an increase in rodent populations, with the highest incidence occurring in Zakarpattia Oblast (12.08 cases per 100,000) [15].

Chamankar's research emphasizes the multifaceted nature of health crises by highlighting the loss of public health in conflict areas due to infectious diseases, poverty, starvation, drug shortages, and war disruptions [16]. According to Petakh et al., the incidence of infectious diseases in 2023 showed mixed results, with viral hepatitis A, chronic hepatitis B, chronic hepatitis C, and tuberculosis experiencing a sharp increase, while rotavirus, salmonellosis, and shigellosis decreased [17]. Zinszer and Abuzerr found that infectious diseases were more prevalent among displaced people, mostly due to insecurity factors such as limited access to sanitary facilities and clean water. Their results demonstrated these communities' resilience and psychological stress [18].

The findings show that violence significantly affects health outcomes and disease treatment, highlighting the challenges faced by displaced persons and the difficulties in allocating healthcare resources. According to Jarman et al., a patient who had been forcibly relocated acquired monkeypox after coming into contact with a large forest rat. Antibiotics were successfully used to treat the patient [19]. To manage public health in conflict-affected areas, Srivastava et al. emphasized strategic planning in resource allocation, with a particular focus on minimizing the burden of illness and desynchronizing epidemic peaks among different population segments [20]. The study by Siddig et al. focused on the challenges faced by a patient who stopped taking folic acid and itraconazole due to war-related obstacles, resulting in ineffective herbal remedies. It emphasized the importance of expanding access to healthcare and resuming treatment through education, especially in areas affected by violence [21].

Nevertheless, this study was marred by a few limitations such as comparative problems, low selection domain scores, and the possibility of bias. Given these limitations, the findings and conclusions might not be comprehensive. To improve the study's findings, several tactics were employed, including removing bias and managing confounding variables. Additionally, numerous studies have demonstrated heightened methodological rigor and an emphasis on successful public health measures, such as vaccination campaigns and WASH improvements. Future studies should examine the relationship between environmental factors and disease incidence, track health outcomes in conflict-affected areas, assess the psychological stress experienced by displaced populations, combine traditional and modern treatments, and review controlled trials with comparable results.

## Conclusions

In conclusion, there exists a complex relationship between conflict and the prevalence, transmission, and management of infectious diseases. Armed conflicts pose significant public health challenges by disrupting healthcare systems, worsening sanitation conditions, and increasing the vulnerability of affected populations to infectious diseases. Improving WASH conditions is critical, given their direct influence on disease transmission and overall health. Future research should investigate the socioeconomic determinants of health, evaluate the efficacy of public health initiatives, and develop strategies for optimal resource allocation. Longitudinal studies are essential to understanding the long-term health effects of war, while research into community resilience and adaptation strategies can inform interventions to better support populations facing ongoing conflict and instability.
